# Assessment of the Effect of Sorafenib on Omega-6 and Omega-3 Epoxyeicosanoid Formation in Patients with Hepatocellular Carcinoma

**DOI:** 10.3390/ijms21051875

**Published:** 2020-03-09

**Authors:** Can G. Leineweber, Anne Pietzner, Ingrid W. Zhang, Usha B. Blessin, Michael Rothe, Eckart Schott, Nils H. Schebb, Karsten H. Weylandt

**Affiliations:** 1 Medical Department B, Division of Hepatology, Gastroenterology, Oncology, Hematology, Palliative Care, Endocrinology and Diabetes, Ruppin General Hospital, Brandenburg Medical School, 16816 Neuruppin, Germany; can.leineweber@mhb-fontane.de (C.G.L.); Anne.Pietzner@mhb-fontane.de (A.P.); 2 Institut d’Investigacions Biomèdiques August Pi i Sunyer (IDIBAPS), 08036 Barcelona, Spain; 3 Medical Department, Division of Hepatology and Gastroenterology, Campus Virchow-Klinikum, Charité Universitätsmedizin, 13353 Berlin, Germany; usha.blessin@charite.de; 4 Lipidomix, 13125 Berlin, Germany; michael.rothe@lipidomix.de; 5 Helios Klinikum Emil von Behring, 14165 Berlin, Germany; 6 Institute of Food Chemistry, University of Wuppertal, 42119 Wuppertal, Germany; schebb@uni-wuppertal.de

**Keywords:** hepatocellular carcinoma, HCC, soluble epoxide hydrolase, sorafenib, EET, EDP, omega-3 fatty acids, n-3 PUFA, arachidonic acid, docosahexaenoic acid

## Abstract

Hepatocellular carcinoma (HCC) is a leading cause of cancer death. The multikinase inhibitor sorafenib is widely used for systemic therapy in advanced HCC. Sorafenib might affect epoxyeicosanoids, as it is also a potent inhibitor of the soluble epoxide hydrolase (sEH), which catalyzes the conversion of epoxides derived from long-chain polyunsaturated fatty acids (PUFAs), such as arachidonic acid (AA) and omega-3 docosahexaenoic acid (DHA), into their corresponding diols. Experimental studies with AA-derived epoxyeicosatrienoic acids (EETs) showed that they can promote tumor growth and metastasis, while DHA-derived 19,20-epoxydocosapentaenoic acid (19,20-EDP) was shown to have anti-tumor activity in mice. In this pilot study, we assessed the effect of sorafenib treatment on the presence of lipid mediators, such as EETs, in blood of the patients with HCC using the lipidomics technology. We found a significant increase in 11,12-EET and 14,15-EET levels in HCC patients treated with sorafenib. Furthermore, while not significant in this small sample set, the data presented indicate that sorafenib can also increase the level of omega-3 DHA-derived 19,20-EDP. While the effect on EETs might hamper the anti-tumor effect of sorafenib, we hypothesize that supplementation of DHA in sorafenib-treated HCC patients could increase the level of 19,20-EDP and thereby enhance its anti-tumor effect.

## 1. Introduction

Hepatocellular carcinoma (HCC) is the third leading cause of cancer death globally [1]. Incidences in the United States and Europe are increasing. With more than 840,000 new cases and more than 780,000 deaths per year worldwide, HCC constitutes a serious health problem [2]. Besides hepatitis B and C [3], and excessive alcohol intake [4], non-alcoholic fatty liver [5] is an important disease predisposing to the development of HCC. When cirrhosis of the liver occurs as a result of chronic liver damage, the risk of HCC increases in parallel with progressive hepatic dysfunction [1].

Limited stages of HCC are treated by surgical resection, liver transplantation, or local ablation procedures (afterloading, transarterial chemoembolization, and others). Depending on severity of the underlying liver disease, survival of patients with HCC is > 5 years (ablation, resection, transplantation) [1].

However, once disease has progressed to more extensive disease, survival is usually severely compromised and is at approximately one year even with systemic therapy [1,6]. Currently, a widely used systemic therapy is the oral multikinase inhibitor sorafenib. Sorafenib predominantly targets the receptor tyrosine kinase of the vascular endothelial growth factor (VEGF) receptors 1, 2, and 3. Furthermore, it targets the RAF/MEK/ERK signaling pathway, specifically the serine-threonine kinases C-Raf and B-Raf [7], and the platelet-derived growth factor receptor-β (PDGFR-β) [8,9]. Sorafenib increases survival in patients with advanced HCC by approximately three months [8]. Lenvatinib, another oral receptor tyrosine kinase inhibitor, was recently approved for the treatment of adult patients with advanced or inoperable HCC. Lenvatinib has a different target profile compared to sorafenib [10], with inhibition of the VEGF receptors 1, 2, and 3, fibroblast growth factor (FGF) receptors 1, 2, 3, and 4, PDGFR-α, and the protooncogenes RET and KIT [9]. Another promising treatment option for at least a subset of patients with advanced HCC is immunotherapy with checkpoint inhibitors. The checkpoint inhibitors nivolumab and pembrolizumab were recently approved for certain patients with HCC by the FDA [9,11].

Sorafenib has also been described to have mild to moderate effects on CYP enzyme functions [12]. However, it is also known as the inhibitor of the human soluble epoxide hydrolase (sEH) [13]. sEH catalyzes the conversion of epoxides derived from long-chain polyunsaturated fatty acids (LC-PUFAs) produced by cytochrome P450 (CYP450) enzymes, such as arachidonic acid (AA), eicosapentaenoic acid (EPA), and docosahexaenoic acid (DHA), into their biologically less active corresponding diols [14,15]. These actions of sorafenib might carry clinically relevant consequences, as the role of epoxy fatty acids in processes such as inflammation and angiogenesis, as well as tumor growth, is well established.

Overexpression of sEH leads to higher metabolization of anti-inflammatory epoxyeicosatrienoic acids (EETs) [16,17] into their biologically less active or proinflammatory diols [17]. Indeed, inhibition of sEH can reduce inflammation [15]. In a high-carbohydrate high-fat diet-induced metabolic syndrome in rats, sEH inhibition controls the metabolic syndrome with alleviation of lipid abnormalities, and decreased cardiovascular and liver abnormalities [18]. In experimental cirrhosis in rats, sEH inhibition can attenuate liver fibrosis and reduce portal pressure [19]. Furthermore, a protective effect of sEH inhibition on a fatty liver by increasing beta-oxidation was shown recently in mice [20]. In methionine- and choline-deficient diet-induced non-alcoholic steatohepatitis in mice, a protective effect of EETs was shown [21]. Interestingly, a study examining epoxy metabolites from different PUFAs found a protective effect particularly of omega-3 derived epoxyeicosanoids in this context [22].

However, EETs were also shown to promote angiogenesis. This proangiogenic effect is mainly due to 11,12-EET and 14,15-EET as a VEGF- and epidermal growth factor (EGF)-dependent mechanism [14]. This proangiogenic effect might explain the finding that EETs have been implicated in tumor growth promotion. Experimental studies have shown that the inhibition of sEH leads to an increase in 14,15-EET, which promotes tumor growth and metastasis by cell invasion [23]. In contrast, experimental studies in mice have shown that an increase in omega-3 DHA-derived 19,20-epoxydocosapentaenoic acid (19,20-EDP) in plasma and tumor obtained by adding a low dose of sEH inhibitors is associated with a decrease in tumor growth by inhibition of tumor angiogenesis and reduced cell invasion [24].

Given that sorafenib has an sEH-inhibitory effect in addition to its tyrosine kinase inhibitory action [8,9,13] and has also been described as a CYP inhibitor [12], the aim of this small pilot study was to assess epoxy lipid metabolites in peripheral blood of HCC patients with and without sorafenib therapy in order to assess whether sorafenib treatment might increase the presence of potentially tumor growth-promoting EETs, as well as of potentially tumor growth-suppressing omega-3 derived EDPs in HCC patients in a routine treatment setting.

## 2. Results

Blood samples from a total of *n* = 6 HCC patients (Table 1) were analyzed in a paired fashion with blood taken without or with concomitant sorafenib treatment (defined as administration of sorafenib twice a day to a total daily dose of 400–800 mg for at least 24 h up to several weeks) and analyzed using lipidomics methods.

**Figure 1:**
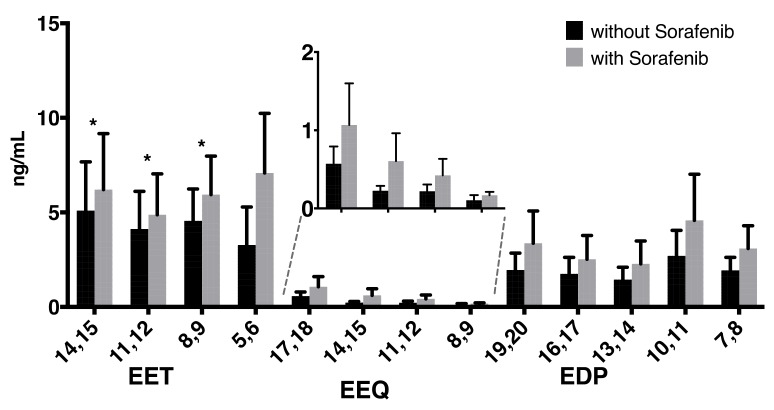
Concentrations of epoxy metabolites without or with concomitant sorafenib treatment. Analysis of epoxy metabolites derived from the omega-6 fatty acid arachidonic acid (AA) (epoxyeicosatrienoic acids, EETs) and from the omega-3 fatty acids eicosapentaenoic acid (EPA) (epoxyeicosatetraenoic acids, EEQs) and docosahexaenoic acid (DHA) (epoxydocosapentaenoic acids, EDPs) in a total of *n* = 6 patients with hepatocellular carcinoma (HCC) receiving sorafenib treatment (* *p* < 0.05).

Levels of EETs (5,6-/8,9-/11,12-/14,15-EET) increased, with significant differences for 8,9-EET, 11,12-EET, and 14,15-EET (Figure 1). Sorafenib treatment also increased levels of epoxy metabolites derived from omega-3 polyunsaturated fatty acids (n-3 PUFAs)—epoxyeicosatetraenoic acids (EEQs) derived from eicosapentaenoic acid (EPA) as well as epoxydocosapentaenoic acids (EDPs) derived from docosahexaenoic acid (DHA), albeit without reaching significance in this small pilot sample. While concentrations of EEQs were much lower than those of the EETs, the EDP metabolite concentrations were approximately half of those observed for the EETs in this group of patients who neither received omega-3 supplements nor dietary advice regarding fish consumption.

Via the sEH, these EETs, EEQs, and EDPs are hydrolyzed enzymatically and become converted into dihydroxy forms: dihydroxyeicosatrienoic acids (DHETs), dihydroxyeicosatetraenoic acids (DiHETEs), and dihydroxydocosapentaenoic acids (DiHDPAs), respectively [14,16]. We found these metabolites to be increased under sorafenib treatment as well (Table 2).

**Figure 2:**
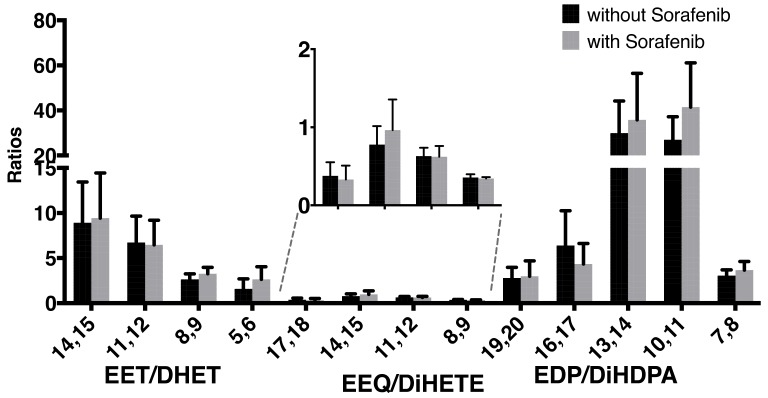
Ratios of epoxy metabolites to their corresponding diols without or with concomitant sorafenib treatment in a total of *n* = 6 patients with hepatocellular carcinoma receiving sorafenib treatment.

As a marker of sEH activity, we then analyzed the ratio of epoxyeicosanoids to their corresponding diols, and particularly of 14,15-EET to 14,15-DHET (Figure 2). As we did not find higher 14,15-EET/DHET ratios, the observed increase in the EETs might not be predominantly due to the sEH-inhibitory effect of sorafenib. The finding that both EETs and DHETs rise under sorafenib treatment shown in Table 2 could point towards an effect on epoxyeicosanoid production, and not on epoxyeicosanoid hydrolysis.

## 3. Discussion

In summary, we were able to demonstrate significant EET increases and a trend towards increased omega-3 epoxyeicosanoids even in this small group of heterogenous patients with hepatocellular carcinoma receiving sorafenib treatment. Despite different ages, different HCC treatments before sorafenib therapy, and various underlying liver diseases, this effect could be observed in a routine clinical setting.

While the sEH-inhibitory effect of sorafenib is well established [13], its effect on the CYP function and expression is less clear. In vitro studies with human liver microsomes have demonstrated that sorafenib is a competitive inhibitor of CYP2C8 and CYP3A isoenzymes [25]. In another study, a weak inducing effect of sorafenib on CYP3A and a modest induction of CYP2C19 activity was observed [12]. Sorafenib may thus have a complex impact on epoxy metabolite formation as observed in this pilot trial. In conclusion, the data in this pilot study point towards increased epoxyeicosanoid formation rather than sEH inhibition as the more relevant mechanism for the observed epoxyeicosanoid increase.

Most of the n-6 AA-derived epoxy metabolites (EETs) are signaling molecules that have anti-inflammatory, vasodilating, antihypertensive, antidiabetic, cardiovascular, renal-protective, proangiogenic, and analgesic effects [26]. The anti-inflammatory effects of 11,12-EET on the endothelium occur by inhibiting IKK and TNF-α activities; this inhibits the expression of adhesion molecules (VCAM-1) [14,27]. Interestingly, it was shown that the corresponding diol DHET, in contrast to the anti-inflammatory EET, has proinflammatory effects [17,26]. This could be evidence to support the anti-inflammatory effects of EETs by stabilizing EETs through sEH-inhibition.

However, as indicated above, EETs could also play a role in tumor progression due to angiogenic effects [23]. The 14,15-EET regioisomer was identified to promote tumor growth and metastasis by cell invasion [14,23]. In addition to 14,15-EET, 11,12-EET promotes tumor angiogenesis by endothelial cell proliferation [28]. Additionally, 5,6- and 8,9-EET were found to increase cell proliferation and de novo vascularization, and thus promote tumor growth [29]. In some tumor tissues, CYP2J2 is upregulated, which forms EETs and increases tumor growth via the proangiogenic effect [14].

The n-3 DHA- and EPA-derived epoxy metabolites (EDPs, EEQs) also have anti-inflammatory, vasodilating, antihypertensive, cardioprotective, and analgesic effects [30,31]. However, in contrast to the EETs, they have an anti-angiogenic effect [14,24]. Experimental studies on mice have shown that an increase in the DHA-derived 19,20-epoxydocosapentaenoic acid (19,20-EDP) in plasma and tumor obtained by adding a low dose of sEH inhibitors is associated with a decrease of tumor growth by inhibition of tumor angiogenesis and reduced cell invasion [24]. Not only VEGF-induced neovascularization, but also fibroblast growth factor (FGF-2)-induced angiogenesis was inhibited [24]. Furthermore, the potential role of EDPs as inhibitors of lymphangiogenesis is currently evaluated, which could further inhibit tumor metastasis [14,32]. This anti-angiogenic effect has also been demonstrated in animal experiments, in which EDPs inhibited tumor growth (breast cancer) and metastasis (lung cancer) [14]. In experiments with cell cultures, the invasive properties of prostate cancer cells were strongly inhibited by EDPs, in contrast to the effect of 11,12-EET [24,33]. In a recent study, besides anti-angiogenic and anti-inflammatory effects, an inhibitory effect of omega-3 epoxyeicosanoids on the RAS/RAF/ MEK/ERK pathway was also described [34].

Tumor growth-suppressing n-3 PUFA-derived epoxy metabolites, such as 19,20-EDP, also showed a tendency to be elevated as a result of sorafenib treatment in the HCC patients studied here, although this effect was not significant in this small pilot study in the patients without DHA supplementation. Stabilization of both tumor-promoting EET and tumor-suppressing EDP metabolites adds strength to the suggestion for supplementary therapy with DHA in HCC patients treated with sorafenib to promote an increase of tumor-suppressing EDP levels [14]. It was shown that CYP450 with EPA and/or DHA as a substrate is an efficient catalyst. Presumably, compared to AA, with EPA or DHA as a substrate, it results in equal or elevated activities [30,35].

Several data sets support a beneficial role of omega-3 fatty acids to suppress HCC development in animal models [36,37,38,39], as well as in humans [40,41,42,43]. The described effect of 19,20-EDP with impact on tumor growth and metastasis might be a mechanism for the protective effect of the n-3 PUFA DHA in this context, and also suggests that stabilizing epoxy FA, as well as nutritional intervention to increase EPA and DHA levels, in combination with sEH inhibition might be able to further decrease tumor progression in comparison with the treatment with sorafenib alone.

Studies with more patients, analysis of fatty acids as well as of lipid metabolites, and of omega-3 fatty acid supplementation will now be necessary to further explore these hypotheses. Analysis of nutritional supplementation is particularly relevant, since HCC patients might have poor absorption due to severe liver disease, which might compromise beneficial effects of DHA supplementation. Furthermore, in order to understand the role of epoxyeicosanoids in the context of HCC, assaying epoxy metabolites in the context of other HCC treatments (lenvatinib) will also be important. Furthermore, given the inflammation-modulating effects described for epoxyeicosanoids, analysis of epoxyeicosanoid profiles in the context of immunotherapy alone or in combination with sorafenib or lenvatinib will be an important goal for future studies of HCC patients.

## 4. Materials and Methods

### 4.1. Patients and Blood Sampling

Patients with advanced HCC on or before starting sorafenib therapy as part of their board-recommended routine therapy were enrolled into the liver clinic at the Charité University Medicine Berlin, Germany. After a written informed consent was obtained, blood (EDTA) was sampled before the first or, in one patient, 5 days after the last administration of sorafenib and in the course of sorafenib treatment. Duration of sorafenib treatment before the second blood sampling varied from 24 h (i.e., 2 doses) to several weeks, and sorafenib was given in daily doses of 200–800 mg/d. In total, we enrolled 6 patients into this pilot biomarker study.

All of the included patients were undergoing routine clinical treatment and started the sorafenib therapy based on the institution’s interdisciplinary tumor board decisions. All the patients had advanced HCC disease and sustained liver function corresponding to Child–Pugh Class A. Choice of treatment was based solely on the treating physician’s assessment. We approached patients for consent to perform additional blood studies for lipidomics analyses. These analyses were approved by the institutional research ethics committee at the Charité University Medicine Berlin (Approval No EA2/121/08), and the study was performed according to the Declaration of Helsinki.

All of the samples were cooled at 4 °C for approximately 3–4 h. Plasma samples were then obtained by centrifugation (10 min, 4 °C, 3500 rpm), and fractions were separated into different vials. All samples (plasma, blood cell components) were stored at −80 °C until further analysis.

### 4.2. Sample Preparation and LC/ESI-MS/MS

Plasma samples were analyzed for epoxy metabolites using the LC/ESI-MS/MS lipidomics technology as described previously [35]. Lipid mediators and deuterated standards used in this study were purchased from Cayman Chemical (Ann Arbor, MI, USA). Materials used for solid phase extraction (SPE), such as sodium acetate, ethyl acetate, acetic acid, and n-hexane were obtained from Carl Roth (Karlsruhe, Germany), and methanol from Merck (Darmstadt, Germany). Additionally, 99% butylated hydroxytoluene (BHT, 2,6-di-tert-butyl-4-methylphenol) was obtained from Acros Organics (Geel, Belgium), and Bond Elute Certify II columns from Varian (Palo Alto, CA, USA) were used. Other solvents, such as methanol (LC-MS-grade) and acetonitrile (HPLC gradient grade), were obtained from Fisher Scientific (Loughborough, UK).

For sample preparation, an internal standard consisting of 15-HETE-d8 (10 ng), LTB4-d4 (10 ng), PGE2-d2 (5 ng), and ice-cold methanol containing BHT (0.1%) was added to the plasma. The pH was adjusted with 1 M sodium acetate buffer containing 5% v/v methanol at pH 6. After centrifugation, the obtained supernatant was added to SPE columns, which were preconditioned with 3 mL methanol, followed by 3 mL of 0.1 mol/L sodium acetate buffer containing 5% methanol (pH 6). The SPE columns were then washed with 3 mL methanol/H2O (50/50, v/v). For elution, 2.0 mL of n-hexane:ethyl acetate (25:75) with 1% acetic acid were used. The extraction was performed with a SUPELCO Visiprep manifold. The eluate was evaporated on a heating block at 40 °C under a stream of nitrogen to obtain a solid residue and stored at −20 °C until LC/ESI-MS/MS analysis was performed.

The residues were dissolved in 70 µL acetonitrile and analyzed using an Agilent 1200 HPLC system with a binary pump, an autosampler, and a column thermostat with a ZORBAX StableBond 3.5 µm, 2.1 mm × 150 mm column using a solvent system of aqueous formic acid (0.1%) and acetonitrile. The elution gradient was started with 10% acetonitrile, which was increased within 10 min to 90% and held there for 10 min. The flow rate was set at 0.4 mL/min, and injection volume was 7.5 µL. The HPLC was coupled with an Agilent 6460 Triple Quad mass spectrometer with an electrospray ionization source. Analysis of lipid mediators was performed with the Multiple Reaction Monitoring in the negative mode.

### 4.3. Statistical Analysis

Statistical analysis was performed using GraphPad Prism 5 Software (La Jolla, CA, USA). The comparison was made using the Wilcoxon matched-pairs signed-rank test. All values are presented as the mean ± SEM. Statistical significance was set at *p* < 0.05.

## 5. Conclusions

In this pilot trial, we investigated the effect of sorafenib treatment on epoxy lipid mediator concentrations in peripheral blood plasma in a small group of six HCC patients. We were able to demonstrate markedly increased epoxy lipid mediator concentrations in the peripheral blood of HCC patients undergoing sorafenib therapy.

Given the anti-tumor effects described in experimental models for the n-3 PUFA-derived epoxy metabolite 19,20-EDP, these data further strengthen the hypothesis that dietary omega-3 PUFA supplementation in addition to sorafenib treatment could lead to higher anti-tumor efficacy due to a higher level of omega-3 epoxyeicosanoids, such as 19-20-EDP.

## Figures and Tables

**Table 1:** Patient characteristics.

Patient	1	2	3	4	5	6
Age	64	71	48	67	61	73
m/f	m	f	m	m	m	m
HCC treatments before sorafenib administration						
Afterloading	+	-	-	-	-	-
Surgical resection	+	-	+	+	-	-
Transplantation	-	-	-	-	-	-
Transarterial chemoembolization	-	+	-	-	-	-
Underlying liver disease						
Alcoholic liver disease	-	-	-	-	+	-
Hepatitis C	+	+	-	-	-	-
Hepatitis B	-	-	+	-	-	-
NAFLD/NASH	-	-	-	+	-	+

Patient characteristics of the *n* = 6 patients receiving sorafenib treatment that were included in this pilot assessment. NAFLD—non-alcoholic fatty liver disease, NASH—non-alcoholic steatohepatitis, m—male, f—female, +—yes, -—no.

**Table 2:** Epoxy and corresponding dihydroxy metabolites in blood samples.

	Without Sorafenib		With Sorafenib		
	ng/ml	SEM	ng/ml	SEM	
14,15-EET	**5.09**	2.58	**6.21**	2.96	*
11,12-EET	**4.11**	2.00	**4.87**	2.17	*
8,9-EET	**4.55**	1.68	**5.94**	2.04	*
5,6-EET	**3.28**	1.83	**7.08**	3.15	
14,15-DHET	**0.53**	0.06	**0.66**	0.12	
11,12-DHET	**0.53**	0.05	**0.73**	0.23	
8,9-DHET	**1.51**	0.26	**1.71**	0.37	
5,6-DHET	**2.51**	0.56	**2.96**	0.71	
17,18-EEQ	**0.57**	0.22	**1.07**	0.53	
14,15-EEQ	**0.23**	0.06	**0.61**	0.36	
11,12-EEQ	**0.22**	0.08	**0.42**	0.21	
8,9-EEQ	**0.11**	0.05	**0.17**	0.03	
17,18-DiHETE	**1.66**	0.21	**3.32**	0.61	*
14,15-DiHETE	**0.30**	0.06	**0.54**	0.11	
11,12-DiHETE	**0.18**	0.04	**0.71**	0.23	
8,9-DiHETE	**0.66**	0.18	**1.39**	0.62	
19,20-EDP	**1.95**	0.90	**3.37**	1.71	
16,17-EDP	**1.75**	0.87	**2.52**	1.26	
13,14-EDP	**1.44**	0.65	**2.28**	1.21	
10,11-EDP	**2.70**	1.35	**4.57**	2.44	
7,8-EDP	**1.93**	0.68	**3.09**	1.21	
19,20-DiHDPA	**0.66**	0.09	**1.28**	0.27	
16,17-DiHDPA	**0.33**	0.03	**0.68**	0.16	
13,14-DiHDPA	**0.05**	0.00	**0.07**	0.02	
10,11-DiHDPA	**0.09**	0.01	**0.09**	0.02	
7,8-DiHDPA	**0.62**	0.14	**0.76**	0.14	

Shown are the means of lipid mediator formation in ng/mL ± SEM (indicated in bold) in *n* = 6 patients with hepatocellular carcinoma without and with sorafenib treatment. Statistical differences were determined using the Wilcoxon signed-rank test (* *p* < 0.05).

## Abbreviations

AA	arachidonic acid
BHT	butylated hydroxytoluene
CYP450	cytochrome P450
DHA	docosahexaenoic acid
DHET	dihydroxyeicosatrienoic acid
DiHDPA	dihydroxydocosapentaenoic acid
DiHETE	dihydroxyeicosatetraenoic acid
EDP	epoxydocosapentaenoic acid
EEQ	epoxyeicosatetraenoic acid
EET	epoxyeicosatrienoic acid
EGF	epidermal growth factor
EPA	eicosapentaenoic acid
FGF	fibroblast growth factor
HCC	hepatocellular carcinoma	
LC-PUFA	long-chain polyunsaturated fatty acid
n-3 PUFA	omega-3 polyunsaturated fatty acid
NAFLD	non-alcoholic fatty liver disease
NASH	non-alcoholic steatohepatitis
PDGFR-ß	platelet-derived growth factor receptor ß
sEH	soluble epoxide hydrolase
SPE	solid phase extraction
VEGF	vascular endothelial growth factor

## References

[1] Forner A., Reig M., Bruix J. (2018). Hepatocellular carcinoma. Lancet (Lond. Engl.).

[2] Bray F., Ferlay J., Soerjomataram I., Siegel R.L., Torre L.A., Jemal A. (2018). Global cancer statistics 2018: Globocan estimates of incidence and mortality worldwide for 36 cancers in 185 countries. CA Cancer J. Clin..

[3] El-Serag H.B. (2012). Epidemiology of viral hepatitis and hepatocellular carcinoma. Gastroenterology.

[4] Morgan T.R., Mandayam S., Jamal M.M. (2004). Alcohol and hepatocellular carcinoma. Gastroenterology.

[5] Sherman M. (2014). Surveillance for hepatocellular carcinoma. Best Pract. Res. Clin. Gastroenterol..

[6] Forner A., Llovet J.M., Bruix J. (2012). Hepatocellular carcinoma. Lancet (Lond. Engl.).

[7] Wilhelm S.M., Carter C., Tang L., Wilkie D., McNabola A., Rong H., Chen C., Zhang X., Vincent P., McHugh M. (2004). Bay 43-9006 exhibits broad spectrum oral antitumor activity and targets the raf/mek/erk pathway and receptor tyrosine kinases involved in tumor progression and angiogenesis. Cancer Res..

[8] Llovet J.M., Ricci S., Mazzaferro V., Hilgard P., Gane E., Blanc J.F., de Oliveira A.C., Santoro A., Raoul J.L., Forner A. (2008). Sorafenib in advanced hepatocellular carcinoma. N. Engl. J. Med..

[9] Mody K., Abou-Alfa G.K. (2019). Systemic therapy for advanced hepatocellular carcinoma in an evolving landscape. Curr. Treat. Options Oncol..

[10] Zschabitz S., Grullich C. (2018). Lenvantinib: A tyrosine kinase inhibitor of vegfr 1-3, fgfr 1-4, pdgfralpha, kit and ret. Recent Results Cancer Res.

[11] Harding J.J. (2018). Immune checkpoint blockade in advanced hepatocellular carcinoma: An update and critical review of ongoing clinical trials. Future Oncol. (Lond. Engl.).

[12] Flaherty K.T., Lathia C., Frye R.F., Schuchter L., Redlinger M., Rosen M., O’Dwyer P.J. (2011). Interaction of sorafenib and cytochrome p450 isoenzymes in patients with advanced melanoma: A phase i/ii pharmacokinetic interaction study. Cancer Chemother. Pharmacol..

[13] Liu J.Y., Park S.H., Morisseau C., Hwang S.H., Hammock B.D., Weiss R.H. (2009). Sorafenib has soluble epoxide hydrolase inhibitory activity, which contributes to its effect profile in vivo. Mol. Cancer Ther..

[14] Zhang G., Kodani S., Hammock B.D. (2014). Stabilized epoxygenated fatty acids regulate inflammation, pain, angiogenesis and cancer. Prog. Lipid Res..

[15] Wang W., Yang J., Zhang J., Wang Y., Hwang S.H., Qi W., Wan D., Kim D., Sun J., Sanidad K.Z. (2018). Lipidomic profiling reveals soluble epoxide hydrolase as a therapeutic target of obesity-induced colonic inflammation. Proc. Natl. Acad. Sci. USA.

[16] Gabbs M., Leng S., Devassy J.G., Monirujjaman M., Aukema H.M. (2015). Advances in our understanding of oxylipins derived from dietary pufas. Adv. Nutr. (Bethesda Md.).

[17] Imig J.D., Hammock B.D. (2009). Soluble epoxide hydrolase as a therapeutic target for cardiovascular diseases. Nat. Rev. Drug Discov..

[18] Iyer A., Kauter K., Alam M.A., Hwang S.H., Morisseau C., Hammock B.D., Brown L. (2012). Pharmacological inhibition of soluble epoxide hydrolase ameliorates diet-induced metabolic syndrome in rats. Exp. Diabetes Res..

[19] Zhang C.H., Zheng L., Gui L., Lin J.Y., Zhu Y.M., Deng W.S., Luo M. (2018). Soluble epoxide hydrolase inhibition with t-tucb alleviates liver fibrosis and portal pressure in carbon tetrachloride-induced cirrhosis in rats. Clin. Res. Hepatol. Gastroenterol..

[20] Yao L., Cao B., Cheng Q., Cai W., Ye C., Liang J., Liu W., Tan L., Yan M., Li B. (2019). Inhibition of soluble epoxide hydrolase ameliorates hyperhomocysteinemia-induced hepatic steatosis by enhancing beta-oxidation of fatty acid in mice. Am. J. Physiol. Gastrointest. Liver Physiol..

[21] Wang X., Li L., Wang H., Xiao F., Ning Q. (2019). Epoxyeicosatrienoic acids alleviate methionine-choline-deficient diet-induced non-alcoholic steatohepatitis in mice. Scand. J. Immunol..

[22] Lopez-Vicario C., Alcaraz-Quiles J., Garcia-Alonso V., Rius B., Hwang S.H., Titos E., Lopategi A., Hammock B.D., Arroyo V., Claria J. (2015). Inhibition of soluble epoxide hydrolase modulates inflammation and autophagy in obese adipose tissue and liver: Role for omega-3 epoxides. Proc. Natl. Acad. Sci. USA.

[23] Panigrahy D., Edin M.L., Lee C.R., Huang S., Bielenberg D.R., Butterfield C.E., Barnes C.M., Mammoto A., Mammoto T., Luria A. (2012). Epoxyeicosanoids stimulate multiorgan metastasis and tumor dormancy escape in mice. J. Clin. Investig..

[24] Zhang G., Panigrahy D., Mahakian L.M., Yang J., Liu J.Y., Stephen Lee K.S., Wettersten H.I., Ulu A., Hu X., Tam S. (2013). Epoxy metabolites of docosahexaenoic acid (dha) inhibit angiogenesis, tumor growth, and metastasis. Proc. Natl. Acad. Sci. USA.

[25] Filppula A.M., Neuvonen P.J., Backman J.T. (2014). In vitro assessment of time-dependent inhibitory effects on cyp2c8 and cyp3a activity by fourteen protein kinase inhibitors. Drug Metab. Dispos..

[26] Morisseau C., Hammock B.D. (2013). Impact of soluble epoxide hydrolase and epoxyeicosanoids on human health. Annu. Rev. Pharmacol. Toxicol..

[27] Node K., Huo Y., Ruan X., Yang B., Spiecker M., Ley K., Zeldin D.C., Liao J.K. (1999). Anti-inflammatory properties of cytochrome p450 epoxygenase-derived eicosanoids. Science (N. Y.).

[28] Yan G., Chen S., You B., Sun J. (2008). Activation of sphingosine kinase-1 mediates induction of endothelial cell proliferation and angiogenesis by epoxyeicosatrienoic acids. Cardiovasc. Res..

[29] Pozzi A., Macias-Perez I., Abair T., Wei S., Su Y., Zent R., Falck J.R., Capdevila J.H. (2005). Characterization of 5,6- and 8,9-epoxyeicosatrienoic acids (5,6- and 8,9-eet) as potent in vivo angiogenic lipids. J. Biol. Chem..

[30] Arnold C., Konkel A., Fischer R., Schunck W.H. (2010). Cytochrome p450-dependent metabolism of omega-6 and omega-3 long-chain polyunsaturated fatty acids. Pharmacol. Rep..

[31] Arnold C., Markovic M., Blossey K., Wallukat G., Fischer R., Dechend R., Konkel A., von Schacky C., Luft F.C., Muller D.N. (2010). Arachidonic acid-metabolizing cytochrome p450 enzymes are targets of {omega}-3 fatty acids. J. Biol. Chem..

[32] Zheng W., Aspelund A., Alitalo K. (2014). Lymphangiogenic factors, mechanisms, and applications. J. Clin. Investig..

[33] Nithipatikom K., Brody D.M., Tang A.T., Manthati V.L., Falck J.R., Williams C.L., Campbell W.B. (2010). Inhibition of carcinoma cell motility by epoxyeicosatrienoic acid (eet) antagonists. Cancer Sci..

[34] Xia R., Sun L., Liao J., Li H., You X., Xu D., Yang J., Hwang S.H., Jones R.D., Hammock B. (2019). Inhibition of pancreatic carcinoma growth through enhancing omega-3 epoxy polyunsaturated fatty acid profile by inhibition of soluble epoxide hydrolase. Anticancer Res..

[35] Fischer R., Konkel A., Mehling H., Blossey K., Gapelyuk A., Wessel N., von Schacky C., Dechend R., Muller D.N., Rothe M. (2014). Dietary omega-3 fatty acids modulate the eicosanoid profile in man primarily via the cyp-epoxygenase pathway. J. Lipid Res..

[36] Griffitts J., Saunders D., Tesiram Y.A., Reid G.E., Salih A., Liu S., Lydic T.A., Busik J.V., Kang J.X., Towner R.A. (2010). Non-mammalian fat-1 gene prevents neoplasia when introduced to a mouse hepatocarcinogenesis model: Omega-3 fatty acids prevent liver neoplasia. Biochim. Et Biophys. Acta.

[37] Lim K., Han C., Dai Y., Shen M., Wu T. (2009). Omega-3 polyunsaturated fatty acids inhibit hepatocellular carcinoma cell growth through blocking beta-catenin and cyclooxygenase-2. Mol. Cancer Ther..

[38] Weylandt K.H., Krause L.F., Gomolka B., Chiu C.Y., Bilal S., Nadolny A., Waechter S.F., Fischer A., Rothe M., Kang J.X. (2011). Suppressed liver tumorigenesis in fat-1 mice with elevated omega-3 fatty acids is associated with increased omega-3 derived lipid mediators and reduced tnf-alpha. Carcinogenesis.

[39] Inoue-Yamauchi A., Itagaki H., Oda H. (2018). Eicosapentaenoic acid attenuates obesity-related hepatocellular carcinogenesis. Carcinogenesis.

[40] Gao M., Sun K., Guo M., Gao H., Liu K., Yang C., Li S., Liu N. (2015). Fish consumption and n-3 polyunsaturated fatty acids, and risk of hepatocellular carcinoma: Systematic review and meta-analysis. Cancer Causes Control.

[41] Koh W.P., Dan Y.Y., Goh G.B., Jin A., Wang R., Yuan J.M. (2016). Dietary fatty acids and risk of hepatocellular carcinoma in the singapore chinese health study. Liver Int. Off. J. Int. Assoc. Study Liver.

[42] Sawada N., Inoue M., Iwasaki M., Sasazuki S., Shimazu T., Yamaji T., Takachi R., Tanaka Y., Mizokami M., Tsugane S. (2012). Consumption of n-3 fatty acids and fish reduces risk of hepatocellular carcinoma. Gastroenterology.

[43] Wen X., Reynolds L., Mulik R.S., Kim S.Y., Van Treuren T., Nguyen L.H., Zhu H., Corbin I.R. (2016). Hepatic arterial infusion of low-density lipoprotein docosahexaenoic acid nanoparticles selectively disrupts redox balance in hepatoma cells and reduces growth of orthotopic liver tumors in rats. Gastroenterology.

